# Emergency Artery Ligation and Distal Revascularization for a Limb-Threatening Brachial Artery Mycotic Aneurysm Rupture

**DOI:** 10.7759/cureus.66010

**Published:** 2024-08-02

**Authors:** Jetinder Singh, Rosnelifaizur Ramely, Wan Zainira Wan Zain, Andee Dzulkarnaen Zakaria, Mohd Nizam Md Hashim

**Affiliations:** 1 Department of Surgery, School of Medical Sciences, Universiti Sains Malaysia, Kota Bharu, MYS; 2 Colorectal Surgery, Hospital Universiti Sains Malaysia, Kota Bharu, MYS

**Keywords:** emergency surgery, revascularization, intravenous drug abuse, mycotic aneurysm, brachial artery

## Abstract

Brachial artery mycotic aneurysms are very rare and even more uncommon to present initially with bleeding or rupture. Initial presentation of ruptured brachial artery mycotic aneurysm in an active intravenous drug abuser is managed with brachial artery ligation with an option of revascularization later. Distal circulation is not commonly threatened as there is a presence of collaterals to perfuse the distal limb. In this case report, we present a case of limb-threatening brachial artery mycotic aneurysm rupture that needed emergency revascularization surgery.

## Introduction

A mycotic aneurysm is a serious medical condition characterized by the presence of an abnormal dilatation of the artery. This is an uncommon form of aneurysm that can arise in any artery in the body. What sets a mycotic aneurysm apart is its association with a fungal or bacterial infection. The infection weakens the vessel wall, making it susceptible to dilation, and can lead to potentially life- or limb-threatening complications if not promptly diagnosed and treated. The presence of mycotic aneurysm in extremities is not common and presenting in a brachial artery is rare.

The usual causes of brachial artery mycotic aneurysms are iatrogenic (vascular access for minimally invasive procedures, blood taking, and cannulation), intravenous drug abuse, and as a consequence of infective endocarditis [1].

Complications of mycotic aneurysms in extremities are bleeding and, to a lesser extent, limb-threatening ischemia. Due to its etiology and risk of infection, rupture of a mycotic aneurysm is treated with emergency ligation and debridement of the wound (when necessary). However, in case of threatened distal circulation, revascularization needs to be considered to avoid amputation in the future [2].

## Case presentation

A 54-year-old man, who was an intravenous drug abuser (IVDA), presented to a hospital nearby with left hand and forearm swelling for a week. There was a necrotic area at the antecubital fossa with surrounding bullae formation as well. Upon initial assessment, there was reduced functionality of the elbow joint due to pain. An ultrasound showed features of left brachial arteriovenous fistula (at the medial antebrachial region) complicated with brachial artery pseudoaneurysm (outpouching with narrow neck seen arising from the left brachial artery measuring 2.4 cm × 1.5 cm) and surrounding hematoma. Subsequently, he started bleeding from his wound site at the antecubital fossa. The patient was then referred to our center.

**Figure 1:**
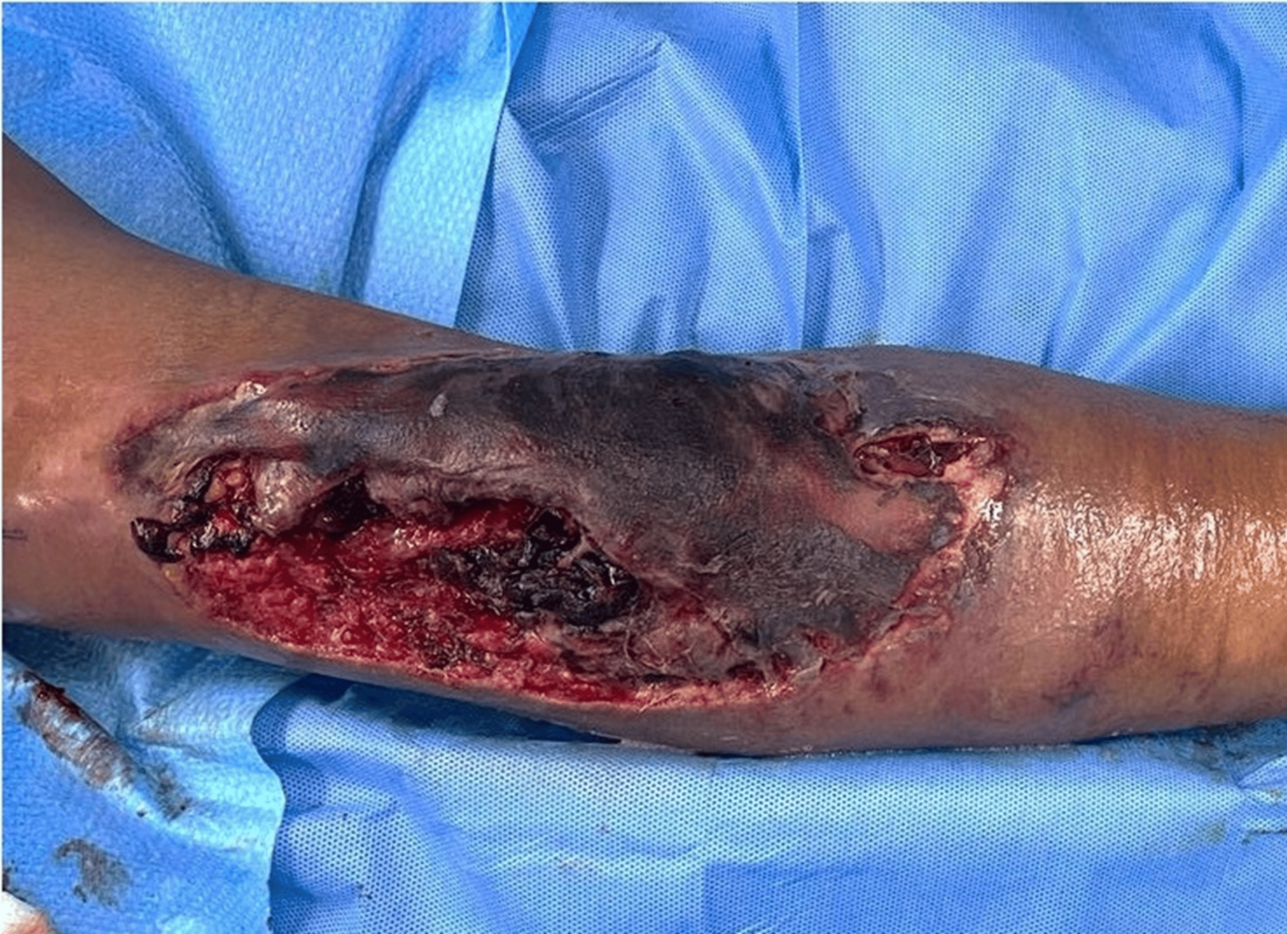
Wound over left antecubital fossa

Upon initial assessment, he was noted to be septic with tachycardia up to 120 beats per minute and a temperature of 38.7°C. He also had generalized swelling over his left upper limb from the elbow joint to his fingers with a large necrotic patch over his proximal forearm involving his antecubital fossa. There was bluish discoloration over the fingertip of his fifth digit. There was also a deep wound over the lateral aspect of the necrotic patch with an active arterial bleed (Figure 1).

A bedside Doppler ultrasound showed a monophasic signal over the distal radial artery and no blood flow over the distal ulnar artery. A biphasic Doppler signal was obtained over the proximal brachial artery above the necrotic patch.

**Figure 2:**
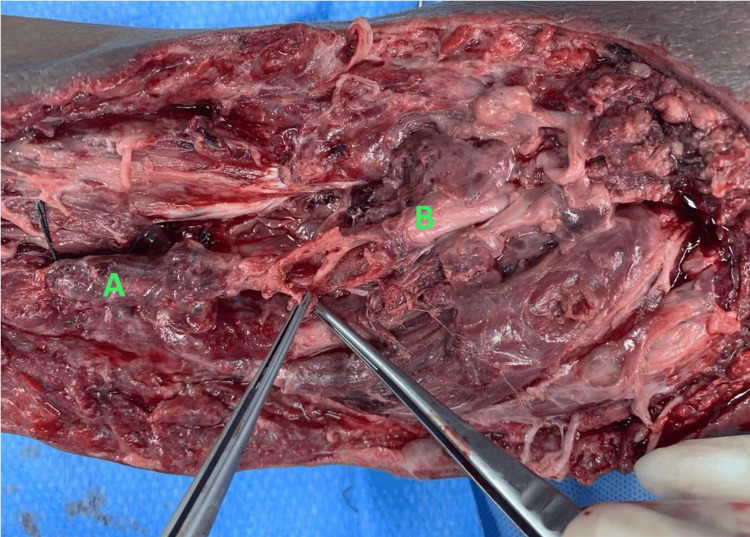
Brachial artery showing ruptured mycotic aneurysm A: Proximal brachial artery. B: Distal brachial artery.

He underwent left brachial artery exploration, ligation, and proximal brachial artery to distal radial artery bypass using the right long saphenous vein graft. Intraoperatively, wound exploration showed grossly infected tissue with some necrotic muscle tissue and a hematoma of about 200 ml. The brachial artery had ruptured just above the cubital fossa with no backflow distally. The brachial artery was then ligated proximally at the proximal edge of the wound, then distal dissection was done until brachial artery bifurcation where the proximal radial and ulnar arteries were ligated separately (Figure 2).

**Figure 3:**
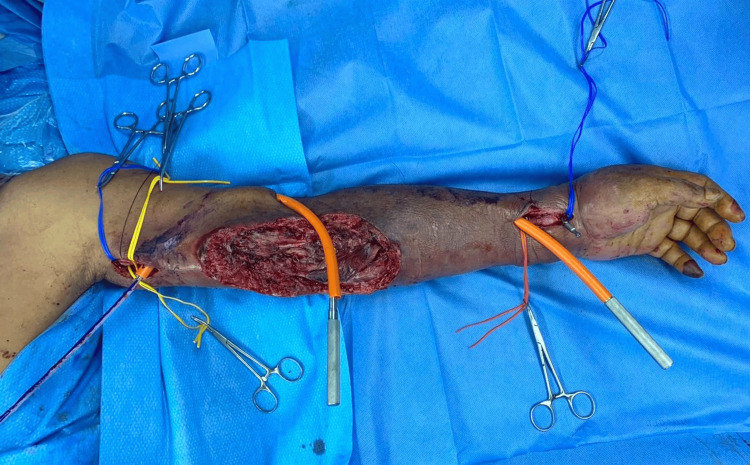
Tunneling of great saphenous vein over the lateral aspect of left upper limb

**Figure 4:**
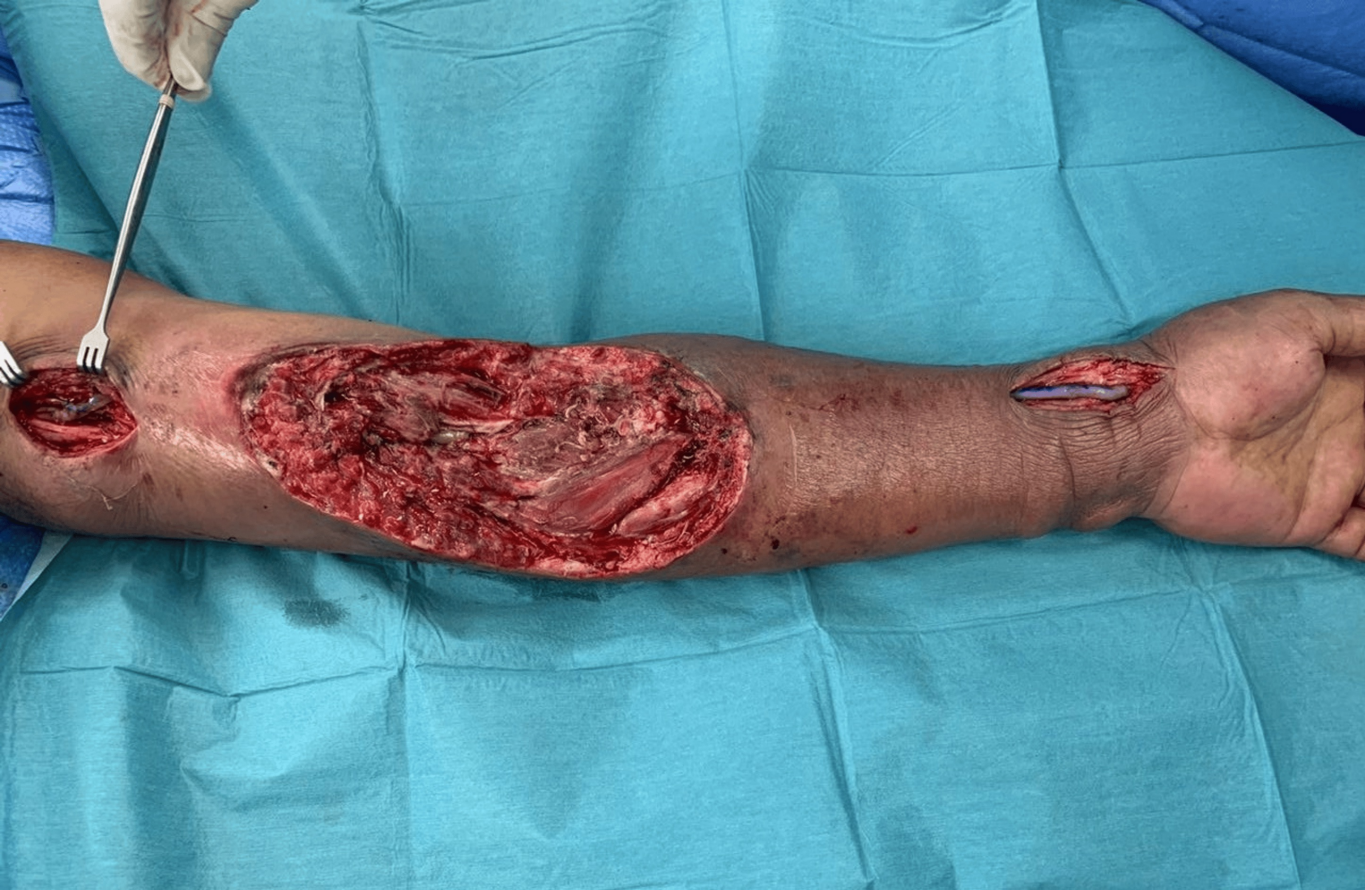
Anastomosis over proximal brachial artery and distal radial artery

A full-length great saphenous vein (GSV) was harvested. The vein was reversed and tunneled subcutaneously along normal (non-infected) tissue lateral to the wound. Anastomosis was done at the proximal brachial artery and distal radial artery (Figures 3, 4).

**Figure 5:**
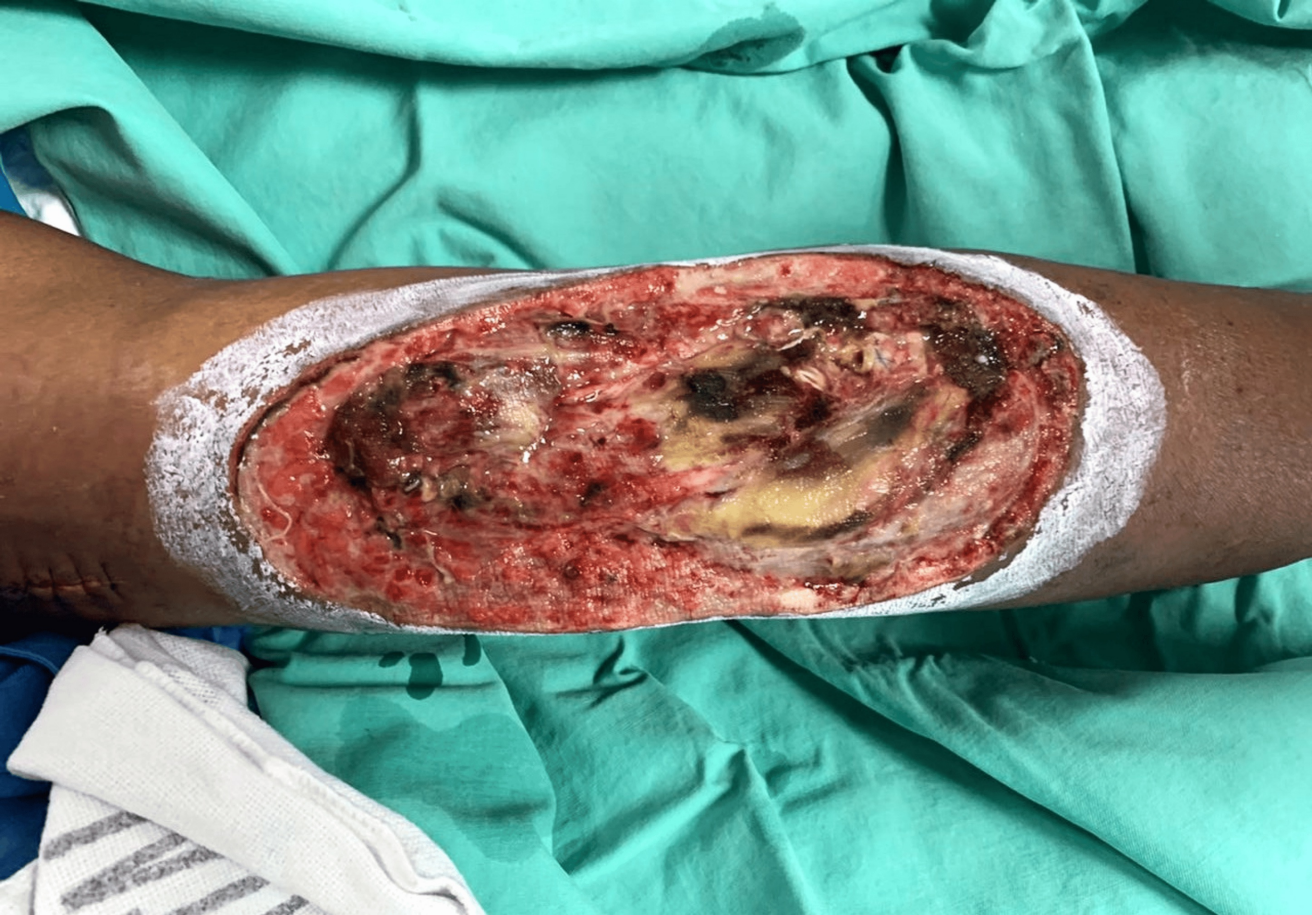
Antecubital fossa wound on day two post surgery

Postoperative examination showed biphasic signal over graft, and distal radius and ulnar arteries. Blood culture, pus culture, and tissue culture grew mixed organisms with the Staphylococcus group predominating in all cultures. The patient was then given antibiotics, wound care, and physiotherapy post surgery. He was discharged home well with outpatient follow-up for his wound (Figure 5).

## Discussion

Mycotic aneurysms of the brachial artery are very rare and are commonly a result of puncture of such arteries. Commonly IVDAs will look for obvious veins for puncture; however, as they do not undergo any training, it is common for them to unknowingly puncture arteries.

Mycotic aneurysms, traditionally known to be caused by bacterial endocarditis, recently have shown more prevalence in IVDAs. These infected aneurysms differ from more common atherosclerotic aneurysms as they are caused by bacterial introduction into the artery through direct puncture. However, these types of conditions can cause a rare but serious complication that can lead to limb loss or even a life-threatening situation. Clinically, these aneurysms are identified by their expanding, throbbing, and painful swelling, often accompanied by redness and induration of the surrounding tissue. These symptoms are typically observed in individuals with a history of intravenous drug use. Physical examination might uncover decreased temperature, a palpable thrill or audible bruit, discoloration, loss of pulsation, and paresthesia in the affected area due to limb-threatening ischemia or, less commonly, nerve compression. Pseudoaneurysms can be distinguished from true aneurysms by the absence of all three layers of the blood vessel walls and by analyzing the waveform in duplex Doppler ultrasound [3]. These aneurysms also will present with local infection and sometimes generalized bacteremia as part of the etiology of the aneurysm if local infection.

In rare circumstances, the initial presentation of these aneurysms can be from embolic or thrombotic events, including ischemia or gangrene needing amputation. These aneurysms have a low risk of rupture and are rarely present in this setting [4]. The patient in this case report presented with a ruptured mycotic aneurysm with threatened distal circulation, which is very uncommon.

The common management approaches for mycotic aneurysms with no distal circulation compromise are brachial artery ligation and debridement of the aneurysmal sac. Brachial artery repair, interposition graft either native or prosthetic, and bypass surgeries are rarely recommended as there is a high risk of infection to the graft causing thrombosis or bleeding. There is usually adequate collateral circulation to provide blood flow distally, so revascularization is rarely needed [1,2].

However, as the patient presented with a limb-threatening condition and distal arm tissue was still viable, the correct decision of bypass surgery was made in the same setting. Brachial artery repair could not be performed as there was extensive tissue infection over the brachial artery mycotic aneurysm site. Interposition graft was also not a viable option as there was inadequate tissue coverage over the bare graft and there is a high risk of infected graft. An autologous saphenous vein graft was chosen as the best option in this situation as the risk of infection and secondary bleeding is lower with native grafts [5]. There are no large-scale studies to show the patency of distal bypass surgery of upper extremities for ruptured mycotic aneurysms.

In previous retrospective studies, it was concluded that revascularization for upper limb ischemia showed high patency rates with native graft (>82%), and limb salvage was achieved in 100% of cases [6]. This is the first surgery of its kind in our center.

## Conclusions

Despite its rare occurrence, it is essential to examine with a high index of suspicion any skin infection in an IVDA patient to rule out aneurysms. Immediate imaging and prompt referral to a vascular center can prevent complications from arising. Revascularization of a threatened limb despite ongoing infection and tissue loss is important for limb salvage.
